# Factors Shaping Attitudes of Medical Staff towards Acceptance of the Standard Precautions

**DOI:** 10.3390/ijerph16061050

**Published:** 2019-03-23

**Authors:** Jerzy Rosiński, Anna Różańska, Andrzej Jarynowski, Jadwiga Wójkowska-Mach, Polish Society of Hospital Infections Team

**Affiliations:** 1 Institute of Economics, Finance and Management, Faculty of Management and Social Communication, Jagiellonian University, ul. Łojasiewicza 4, 30-348 Kraków, Poland; jerzy.rosinski@uj.edu.pl; 2 Department of Microbiology, Faculty of Medicine, Jagiellonian University Collegium Medicum, ul. Czysta 18, 31-121 Kraków, Poland; mbmach@cyf-kr.edu.pl; 3 Faculty of Physics, Astronomy and Applied Computer Science, Jagiellonian University, ul. Łojasiewicza 11, 30-338 Kraków, Poland; ajarynowski@gmail.com; 4 Interdisciplinary Research Institute in Wroclaw, ul. Oriona 15/8, 67-200 Głogów, Poland

**Keywords:** infection control, standard precautions, hand hygiene, healthcare workers behaviour

## Abstract

Standard precautions (SPs) guidelines are the minimum infection prevention practices that apply to all types of patient care, regardless of suspected or confirmed infection status of the patient. They are based on risk assessment, make use of common sense practices and personal protective equipment that protect healthcare providers from infection and prevent the spread of infection from patient to patient. The aim of this study was to determine medical staff’s attitudes towards SPs and analyse the factors shaping these attitudes. The study was conducted using a questionnaire that comprised 25 statements describing the attitudes of medical personnel towards SPs. They were designed to pinpoint the factors that determine these attitudes. There were five factors identified that shape employees’ attitudes towards SPs: assessment of the situation, favourable patterns of behaviour, negative norms, unfavourable patterns of behaviour and rationalising. The study analysed 505 questionnaires filled in by hospital workers from five Polish cities. The majority of the respondents were women (92.1%), nurses (87.5%); the average age was 41.8 and the average seniority was 19.2 years. Over one-third of the respondents worked in non-surgical (36.4%) and surgical (31.6%) wards, 12.3% were employed in intensive care units (ICUs) and 8.9% in emergency departments (EDs). The variable significantly affecting the level of acceptance of SPs was seniority: initially the support was high, then it later decreased, with the greatest decrease occurring between the third and eighth year of work. The staff of medical wards and ICUs demonstrated significantly lower support for SPs and strong environmental impact on SPs perception; low degree of acceptance among medical ward staff correlated negatively with factors from the category “favourable patterns of behaviour”. The substantially strongest support for SPs was found in ED workers. The results indicate the need for continuous education of individual groups of workers concerning the application of SPs, but also the necessity to change the organisational culture in Polish hospitals.

## 1. Introduction

Contemporary medicine offers patients numerous advanced methods for diagnostics and treatment of diseases, and worldwide there has been increased spending on healthcare in recent years. Indeed, the global spending on healthcare increased from 8.6% GDP in 2000 to 10.0% GDP in 2014 [1]. At the same time, in Poland, healthcare expenditure grew, respectively, from 5.3% to 6.35% GDP [1]. However, due to, among other reasons, the application of advanced invasive treatment methods and diagnostics, the use of healthcare services involves the risk of developing healthcare-associated infections (HAIs), including the ones caused by multidrug resistant microorganisms. It is estimated that 5–15% of patients develop infections, the course and consequences of which can be severe [2]. In response to this problem, in the United States in the 1970s, the era of organised, evidence-based infection control began [3]. The basis for the prevention of infections is hand hygiene (HH) [4], which is an element of the so-called standard isolation required for each contact with the patient. Standard precautions (SPs) guidelines are the minimum infection prevention practices that apply to all types of patient care, regardless of suspected or confirmed infection status of the patient. They are based on risk assessment, make use of common sense practices and personal protective equipment that protect healthcare providers from infection and prevent the spread of infection from patient to patient. At times, SPs should be complemented by components of expanded isolation, depending on the state of colonization or infection of the patient with a specific type of microorganism (e.g., in a patient colonized with methicillin-resistant *Staphylococcus aureus* or *Clostridium difficile*, contact isolation should be additionally introduced) [5]. Failure to comply with the precautions requirements, including hand hygiene, can result in the transmission of pathogens and also the contamination of the touch surfaces of hospital wards. The presence of microorganisms causing HAIs on the touch surfaces in patient zones has been confirmed in numerous studies, including Polish studies [6,7]. The traditional and modern methods of cleaning and disinfection serve to ensure a satisfactory level of hygiene in the hospital environment [6,8]. However, compliance with the standard precautions guidelines by medical staff plays a key role in this area.

Although HH, or broadly SPs, is the simplest and cheapest method of preventing infections, the praxis reveals that these principles are often overlooked or applied selectively, in various situations, by different groups of medical workers, both in Poland and in other countries [9,10,11,12]. Polish observational studies on HH compliance among healthcare workers revealed that in the situation prior to contact with the patient only 16.8% of doctors and 4.7% of nurses performed hand hygiene according to the recommendations, and after contact these proportions were 53.1% and 25.3%, respectively [9,10]. These results are very worrisome, because the compliance rates are much lower than in other European countries. Based on an observational study, Ceriale et al. reported that the HH compliance rate achieved by all healthcare workers was 76.84%, and, contrary to a cited Polish study, observed better compliance rates among nurses than doctors [13]. The infection control in Polish healthcare settings continues to be an issue with significant associated challenges and demands additional studies [14].

The objective of this study was to assess the attitudes of different groups of medical staff towards the application of SPs in practice while considering demographic and professional factors that could determine these attitudes, such as age, seniority, place of work, and medical occupation.

## 2. Material and Methods

The research tool employed in our study was an anonymous questionnaire developed by Dr. Stephane Bouchoucha (questions are presented in Table S1 the supplementary file), translated by the authors into Polish, concerning the attitudes of respondents to the application of SPs by medical workers in Polish hospitals. Alongside the questionnaire, the respondents also received a description of the principles of standard isolation, which indicated the need for HH and the application of basic personal protective equipment, especially protective gloves, in situations that require such actions. The body of the questionnaire comprised 25 statements describing the attitudes of medical personnel towards SPs. They were designed to pinpoint the factors that determine these attitudes. Each statement required the respondents to select one of the following possibilities: “not at all”, “a bit”, “neither yes nor no”, “rather yes”, or “very much so”. 

The study made use of a division into five categories, consistent with the specificity of Polish hospitals, with the aim of obtaining an optimal empirical system of dependencies between questions (maximizing intragroup cohesiveness and minimizing intergroup correlations). They were as follows:

1. Assessment of the situation—this factor refers to an employee’s decision whether and to what degree it is appropriate to apply SPs in a given situation; this assessment is made independently by the employee and is based on a subjective estimation of the risk related to performing the task (questions 2, 5, 14–16, 23).
2. Favourable patterns of behaviour—this factor is associated with social influence on the employees’ behaviour in terms of organisational behaviour favourable for SPs; observing the behaviour of other people causes us to want to modify our own behaviour; we also influence the behaviour of our co-workers through the patterns of our own behaviour, sharing knowledge, giving feedback (questions 1, 3, 7, 9, 10). It is a conscious shaping of the attitudes of others in the direction that we consider to be right, while at the same time, we are convinced we are right and try not to give into the negative influence of other people.
3. Negative norms—this factor indicates unfavourable informal rules existing in the organisation, such as group norms, habits and behaviours providing the permission to ignore the need for the application of SPs (questions 11, 12). It is a level of behavioural regulation not disclosed in organisations (usually in organisations we do not speak directly about informal principles or habitually sanctioned norms of behaviour, we rather refer to formal rules and regulations of procedures); this level of behaviour regulation becomes clear when we have to overstep the unwritten, informally existing rules—then, their regulatory force is revealed.
4. Unfavourable patterns of behaviour—this factor is associated with social influence on the employees’ behaviours in terms of inconsistent and/or explicitly negative behaviours; if the majority of people behave in a particular way, we are also inclined to behave the same way. With respect to considerations surrounding the application of SPs, if the majority of people in an organisation apply SPs then the workers themselves apply SPs because it is a standard of behaviour in a given organisation; if inconsistent or explicitly negative behaviours occur, the employees will not be as likely to comply with SPs (questions 4, 6, 8, 13, 24).
5. Rationalising—this factor describes an employee’s tendency to justify his or her own negative behaviour; the worker is focused on “clearing themselves of accusations”; there is no intention to change the unfavourable behaviour patterns (questions 17–22, 25).

Subsequently, using the empirical results of the questionnaires, automatic partitions were determined using principal components and k-means. Based upon knowledge regarding the quality of matching of the partition models and their degree of association (partial correlation method and normalised k-means), with the use of brainstorming (via the authors’ expert assessment), the final set of categories was formed.

Statistical analysis of the data considered the five factors mentioned that shape attitudes, as well as the summary index of support for SPs (overall concept). In the overall concept, an attempt was undertaken to explain which factors have influence on the summary index or the partial index of support for “SPs”. To this end, support indexes towards SPs were defined. Every question—coefficient—was scored a value of support from 1 to 5 (Likert interval scale) depending on the answer obtained on a nominal scale from “not at all” to “very much so”. The main dependent variable analysed here was the summary support towards SPs (Index Sum) as the sum of all 25 statements (recoded answers were directed positively—the point values for questions about statements opposing SPs were opposite to the basic primary scale). Additionally, partial indexes for groups of questions were calculated (according to reference data) and determined by different classification methods. Reference variables (partial indexes) correspond to indirect factors that were not measured directly, which could explain the phenomenon of support towards SPs. Using covariance and regression analyses, the contribution of individual independent variables on dependent variables was determined—indexes of support towards SPs.

In the case of the summary index, a linear regression model was employed, and in the context of partial indexes calculated for individual categories of questions, a logistic regression was applied, and hence, coefficients with variable values were given in relation to the established reference level. The level of *p*-Value < 0.05 was chosen to claim significant relations.

The questionnaire was distributed personally by the authors among the staff in hospitals in which the authors were working and during scientific conferences among workers of hospitals located in five cities of central and southern Poland. The general part (respondents’ particulars) of the questionnaire included the following information: age, sex, seniority, profession, place of work specified as four options: medical wards, surgical wards, intensive care units (ICUs) and emergency departments (EDs).

The study comprised 512 medical workers. Following initial selection, seven questionnaires were excluded from analysis as they were lacking answers to two or more questions regarding the respondents’ particulars. Among 505 surveys analysed, 451 (89.3%) contained answers to all the questions about SPs. A detailed analysis was performed for the most-represented professions: nurses, physicians and paramedics.

The study received approval from the Bioethics Committee of Jagiellonian (No. KBET/122.6120.124.2016)

## 3. Results

Out of 505 questionnaires analysed, 465 (92.1%) were filled in by women and 39 (7.7%) by men (Table 1). The respondents represented various medical professions, including 442 nurses (87.5%), 19 physicians (3.8%), 32 paramedics (6.3%), and 10 others (2%), including laboratory assistants or dental technicians. The average age of the respondents amounted to 41.8 years (standard deviation (SD) 9.47) and was significantly higher for women than men (42.6 years (SD 9.06) vs. 32.0 years (SD 8.90), respectively). Similarly, gender differences were observed with regard to work experience, with the women typically having more years of work experience than the men (20.1 years of work (SD 10.21) vs. 7.6 years of work (SD 7.33), respectively). The largest group among the respondents were people working in medical wards (36.4% of the total), and surgical wards (31.6%). Only 12.3% of respondents worked in ICUs and only 8.9% worked in emergency departments (Table 1).

The elements significantly impacting the acceptance/negation of the principles of SPs application were the place of work and seniority. Working in medical wards had a significant negative influence on support for SPs Similarly the influence of working in ICU was negative, although insignificant (however borderline), whereas working in ED wards had a significant positive influence (Table 2, Table 3).

**Figure 1:**
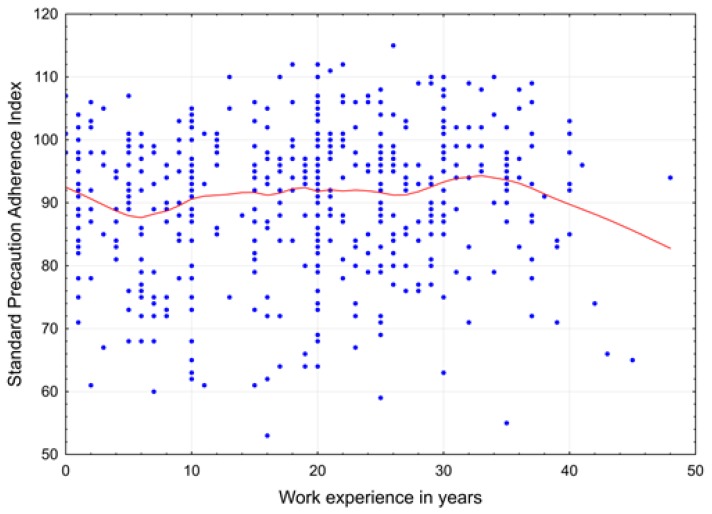
Impact of work experience on Sum Index of support towards SPs depending on respondents’ age.

Regardless of the place of work and profession of the subjects studied, a characteristic (nonlinear) relationship was discovered between work experience and the summary index of acceptance towards SPs (Figure 1) in which every additional year of work experience significantly increases the summary index of support for SPs by 0.17 points. The observed trend is significant: the initial high level of support derived from the period of formal learning of the profession decreased in subsequent years of professional work, with the greatest decrease occurring between the third and eighth year of work (Figure 1). This decline is significant in comparison with the rest of the test set (Student’s *t*-test for means for Sum Index, *p* = 0.002). However, after the initial decrease, a significant increase in support was found (linear regression value of 0.22+/0.08) in workers with 6–34 years of work experience (Figure 1), with support levels subsequently beginning to decrease again, albeit insignificantly (*p* > 0.87), from the 35th year of work. It should be noted, however, that the decline in support for SPs coincides with a decrease in the size of the cohorts.

An analysis concerning the direction of impact of selected categories—adjusted to the socio-cultural conditions of Polish hospitals—on the perception of the application of SPs (Table 4) indicated the fact that the place of work significantly influenced the perception of SPs. A low level of support was significantly more frequently declared by workers of non-surgical wards—the influence of *rationalising*. In contrast, ED workers demonstrated a positive correlation with the *rationalising* factor and a negative correlation with *negative patterns*. The factor *favourable patterns of behaviour* turned out to be the factor significantly more often correlated with lack of support for SPs demonstrated by men and non-surgical ward personnel, and with support for SPs among physicians and surgical ward personnel. The factor *unfavourable patterns of behaviour* turned out to be the factor significantly correlating with support for SPs among workers with longer seniority and surgical ward personnel. As for *assessment of the situation*, none of the independent variables demonstrated statistically significant positive or negative relationships. 

## 4. Discussion

In modern medicine, a particular risk of HAIs is associated with the development of new medical technologies, application of complex medical procedures, growing resistance to antibiotics or treatment of patients of extreme age with multiple comorbidities. Hence, of particular importance are the knowledge, skills and actions of medical personnel with respect to HAI prevention and one of its elements is the use of SPs, especially medical staff’s HH [4,5]. Unfortunately, observing the rules of its proper execution poses a global problem due to low rates of compliance [12]. Data from Poland are also disturbing since as many as 75% of the physicians and medical students examined could not properly perform the Ayliffe handwashing technique that is recommended by the WHO [15]. Most of them attached greater importance to the use of diagnostic gloves than HH [16]. The personnel did not accept the patient as a partner who could remind them of HH, while the patients were reluctant to do so and it was rare that it occurred [17]. There are numerous non-medical (e.g., cultural) difficulties in the implementation of HH principles (e.g., among the Polish nurses and midwives, as many as 40% wear long nails at work, which are also varnished) [18]. Regrettably, the results of this study suggest big problems associated with the practical implementation of the principles of standard isolation, as none of the medical worker groups studied demonstrated full support for their application.

Significant factors of support for SPs principles emerged as, primarily, place of work (type of hospital ward) and seniority. However, it is worth taking into account that factors such as social role (working as a physician) and sex could have modified the results (differentiating variables in the category ‘favourable patterns’) significantly due to the social approval variable [19,20,21]. This means that in the case of the variables mentioned, one should also consider other sources of variation, such as the degree to which an individual might manage the impression that he or she makes on other people (*Impression Management*), either through highlighting one’s own positive qualities (*Self-Deceptive Enhancement)* and/or denying one’s own negative qualities (*Self-Deceptive Denial)*. These additional sources of variation could have disrupted the final results; hence, this study is focused on discussing the results related to the change in support for SPs over time—the influence of seniority—and to the variability of questionnaire factors for the place of work—individual wards, in which, with a high probability, there are no modifying factors associated with the social approval variable.

Research results can be analysed appropriately across all levels of organization: a medical facility as a whole, a team of people working together in a ward, and according to individual employees (physicians, nurses, paramedics), because it is at these levels (organization, team, employee) that possible interventions to increase support for SPs should be considered. 

For a hospital, as an organisation, the observed unfavourable patterns of behaviour can result from perceiving specific events on the basis of *social proof*, which comes down to the mechanism: “*others do it, so I do the same”* [22]. This mechanism would only explain the initial decline in support for SPs in newly employed people: “*I don’t know what work is like here in practice—I will observe it and adapt to the situation.”* It would be an adaptation to the new situation by imitating the existing behaviours, even against the previously learned rules.

Unfortunately, the situation seems to have been more complex, as apart from observing behaviours, social norms related to the regulation of accepted and unaccepted behaviours in the studied work environment also surfaced [23], which came down to the following mechanism: “*if others think so, then I want to be accepted: I will perceive the situation and express my judgments in the same way as others*”. This mechanism does not only concern new situations, it also applies over longer periods. Therefore, unfortunately, the intervention that is probably necessary cannot only concern patterns of behaviour, but also the norms regulating them. 

It may also turn out that the observed disregard of SPs has its source in the problems of the organization that are hidden deeper, such as a lack of involvement on the part of the employees in the hospital’s mission or a lack of decision-making or clearly defined areas of responsibility and competence. In this situation, the dysfunction established at the internal level can effectively disrupt subsequent external levels, although the research material discussed here does not provide a basis for such far-reaching conclusions. 

However, it was not the organisation (hospital) that had a significant impact on the level of acceptance of SPs. A strong influence was associated with the team. Significant differences were found between individual types of hospital wards: ED workers set themselves apart significantly positively. This strong relationship is indicated not only by varying attitudes towards SPs—negative attitudes in non-surgical departments and in intensive care units, in contrast to the positive attitude of employees of surgical wards and EDs—but also by the analysis of the impact of the five categories of factors established. In four out of five cases examined, there was a significant (either positive or negative) correlation with the place of work. Therefore, if the culture generated by individual wards may bring about both positive as well as negative attitudes towards SPs, the presented results show that mainly negative ones are generated in Polish hospitals, and this mainly applies to Polish medical wards and ICUs.

Certainly, it can be assumed that every ward is a separate organisational unit with its own organisational culture, but at the same time, the results presented also show a negative correlation between seniority and SPs. This relationship is nonlinear (Figure 1): while, in the initial period of working in the team, the knowledge obtained during the formal education strengthened the acceptance of SPs, the beginning of stable functioning in the work team caused dissonance between the cognitive component (school education) and the behavioural component (behaviour of co-workers). Additionally, if this discrepancy between the declared knowledge and the behaviour implemented is accompanied by a negative emotional component, what may appear in the employee’s attitude is not only a lack of acceptance, but even negative emotions towards SPs. Unfortunately, combining the dissonance between knowledge and behaviour with simultaneous experience of negative emotions can lead to a faster professional burnout. Luckily, together with growing seniority, there was a significant increase in acceptance for SPs. Therefore, it is possible to conclude that people with long work experience in the ward do not transfer their professional skills to the employees who are just starting out, including the knowledge of SPs, or the scope of the skills is not attractive for the workers with less experience. Perhaps the reason behind it is the conviction that, in medical expertise, there is no room for SPs procedures, although the attitude towards SPs among experienced employees with long work experience is positive (Figure 1), and therefore SPs procedures constitute the area of skills that is not explicitly associated with “being an expert” in a given field.

## 5. Conclusions

Among the staff of medical wards and intensive care units, low acceptance of SPs and strong environmental influence of the team on their perception were exposed, which, together with the frequent omission or disregard for HH rules in Polish hospitals, may involve a high risk of patient and worker exposure to infection. A medical staff’s continuous education should mandatorily entail elements concerning the application of SPs and their components, including HH, regardless of the place of work or medical profession, but especially among the staff with longer work experience (i.e., several years of working experience). Simultaneously, hospitals should take an active role in promoting compliance of SP procedures for senior medical staff with 10–20 years of practical experience.

### Statistical Limitations

Due to the numerous missing answers to the questions from the main part of the questionnaire showing collective properties (e.g., question no. 8 had a significantly different response rate from the rest of the questions), the treatment of deficiencies of data may influence the interpretation of statistical results. With this said, in a control scenario in which it was decided not to reject all incomplete questionnaires, it was the influence of seniority on the index of support towards SPs that would lose statistical significance and the statistical significance of a negative association between belonging to ICU and the index of support for SPs would shift from borderline to appropriate. Moreover, there were difficulties in model matching and selection for partial indexes in operational calculations due to the instability of indexes.

## Figures and Tables

**Table 1:** The demographic characteristics of the study group and support/acceptance for applying the rules of standard isolation precautions (SPs).

Variable	Characteristics of the Study Group		
	Average Age, Years (SD)	Average Seniority, Years (SD)	Average Support for SPs Index Sum * (SD)
**Gender**			
female, *n* = 456	42.6 (9.07)	20.1 (10.21)	90.8 (11.10)
male, *n* = 39	32.1 (8.90)	7. 6 (7.33)	88.1 (11.97)
nd, *n* = 1	50.0 (n/a)	30.0 (n/a)	63.0 (n/a)
**Place of work**			
medical wards, *n* = 184	42.1 (9.06)	19.7 (10.38)	88.7 (12.32)
intensive care units, *n* = 62	42.2 (8.96)	19.2 (10.11)	89.2 (12.36)
surgical wards, *n* = 160	42.2 (8.50)	19.8 (9.33)	92.7 (9.50)
emergency department, *n* = 45	36.2 (12.29)	12.7 (13.01)	92.2 (9.04)
others, *n* = 6	26.0 (n/a)	4.0 (n/a)	87.0 (n/a)
nd, n = 48	46.0 (7.78)	23.5 (9.52)	91.1 (11.88)
**Occupation**			
nurses, *n* = 442	43.2 (8.50)	20.8 (9.67)	90.8 (11.32)
physicians, *n* = 19	39.1 (10.10)	13.3 (9.96)	92.5 (10.51)
paramedics, *n* = 32	27.4 (5.45)	3.6 (3.78)	89.1 (9.44)
others, *n* = 20	26.0 (n/a)	4.0 (n/a)	87.0 (n/a)
nd, *n* = 5	37.8 (9.15)	14.2 (9.58)	76.0 (14.46)
Total, *N* = 505	41.8 (9.47)	19.2 (10.57)	90.5 (11.24)

* Index Sum: support for SPs rules, in total; nd: no data.

**Table 2:** Impact of selected measurable descriptors on the support index for SPs (the analysis was conducted using linear regression).

Variable		Support for SPs Index Sum	
		Power of Impact	*p*-Value
**Age**		−0.10	0.24
Seniority		0.17	0.02
Sex	female	3.44	0.31
	male	0.22	0.95
Profession	nurses	1.12	0.70
	physicians	5.15	0.13
	paramedics	−0.36	0.92
Place of work	medical wards	−2.47	0.01
	intensive care units (ICUs)	−2.48	0.05
	surgical wards	1.47	0.13
	emergency department (ED)	3.76	0.04

**Table 3:** Strength of impact of selected measurable descriptors on the support index for SPs (analysis of variability).

Variable	Test of Significance of Differentiation of Support for SPs, Index Sum		
	degrees of Freedom	MS—Strength of Contribution	*p*-Value
Age	1	4.30	0.84
Seniority	1	77.45	0.39
Sex	2	126.94	0.30
Profession	4	92.42	0.48
Place of work	4	632.15	<0.001

**Table 4:** Diversification of the impact of selected measurable descriptors on partial indexes of support towards SPs (reference levels: sex—female; occupation—nurses; place of work—ICUs. The (+/−) symbol indicates the direction of influence from the analysis of covariance.

Partial sums	Statistically significant dependent variables of influence
(1) Rationalising	non-surgical wards (−) ED (+)
(2) Favourable patterns of behaviour	men (−) physicians (+), non-surgical wards (−) surgical wards (+)
(3) Negative norms	ED (−)
(4) Unfavourable patterns of behaviour	seniority (+), surgical wards (+)
(5) Assessment of the situation	No impact

## Supplementary Materials

The following are available online at https://www.mdpi.com/1660-4601/16/6/1050/s1, Table S1: Factors Influencing Adherence to Standard Precautions Scale ©.
