# Supplementary material for: Factors Shaping Attitudes of Medical Staff towards Acceptance of the Standard Precautions
Source: Int J Environ Res Public Health. 2019 Mar 23;16(6):1050. doi: 10.3390/ijerph16061050 (PMC6466440; doi:10.3390/ijerph16061050)
Supplement: Supplementary file 1 [file ijerph-16-01050-s001.pdf]

Factors Influencing Adherence to Standard Precautions Scale ©

The following questions ask about your use of Standard Precautions in the clinical setting. Please select the answer that best describes you. Please answer all questions.

Question	Not at all	A little	Somewhat	Quite a bit	Very much
**1**. I feel the need to confront people I see not adhering to standard precautions	☐	☐	☐	☐	☐
**2**. The more experienced I become at my job, the more likely I am to be able to decide when I need to use standard precaution	☐	☐	☐	☐	☐
**3**. I am more likely to wear Personal Protective Equipment if I see my colleagues wearing them	☐	☐	☐	☐	☐
**4**. People interpret standard precaution guidelines differently	☐	☐	☐	☐	☐
**5**. I assess what is wrong with a patient before deciding whether or not to implement standard precautions	☐	☐	☐	☐	☐
**6**. If I know that my workplace has a culture of adhering to standard precautions, I am more likely to adhere	☐	☐	☐	☐	☐
**7**. When I witness others' non-adherence with standard precautions, I use that as an education opportunity	☐	☐	☐	☐	☐
**8**. In some workplaces it is standard practice not to follow guidelines	☐	☐	☐	☐	☐
**9.** I use role-modelling to increase use of standard precautions by others	☐	☐	☐	☐	☐
**10**. I have a responsibility to encourage people to protect themselves	☐	☐	☐	☐	☐
**11**. The culture of my organisation allows for people not to follow standard precaution guidelines	☐	☐	☐	☐	☐
**12**. I feel comfortable challenging nurses or doctors when I see them not adhering to standard precautions	☐	☐	☐	☐	☐
**13**. Most nurses typically adhere to standard precautions	☐	☐	☐	☐	☐
**14**. My assessment of a patient's status will indicate if I need to follow standard precautions guidelines	☐	☐	☐	☐	☐
**15**. It is my choice to not wear gloves when taking blood/cannulating as I am only putting myself at risk	☐	☐	☐	☐	☐
**16**. I am able to decide whether or not to use Personal Protective Equipment based on the clinical risks to me	☐	☐	☐	☐	☐
**17**. I am clumsier when I wear gloves and risk having to repeat the procedure	☐	☐	☐	☐	☐
**18**. I am more likely to wear Personal Protective Equipment if they are located nearby patients	☐	☐	☐	☐	☐
**19**. I am more careful if I know that a patient has a blood borne pathogen	☐	☐	☐	☐	☐
**20**. I am more likely to follow standard precautions if I am dealing with needles	☐	☐	☐	☐	☐
**21**. I don't wear gloves as I cannot feel veins	☐	☐	☐	☐	☐
**22**. I am less likely to wear gloves as I was taught procedures without them	☐	☐	☐	☐	☐
**23**. I am educated and able to weigh the risks/benefits of not using standard precautions when needed	☐	☐	☐	☐	☐
**24**. Most doctors typically adhere to standard precautions	☐	☐	☐	☐	☐
**25**. I don't need to wear gloves when taking blood/cannulating as I am skilled at what I do	☐	☐	☐	☐	☐

For further information, permission to use the scale and scoring details with factor distribution please email: s.bouchoucha@latrobe.edu.au

Copyright Stéphane L. Bouchoucha & Kathleen A. Moore (2014)
